# Exploring the Relationship Between Privacy and Utility in Mobile Health: Algorithm Development and Validation via Simulations of Federated Learning, Differential Privacy, and External Attacks

**DOI:** 10.2196/43664

**Published:** 2023-04-20

**Authors:** Alexander Shen, Luke Francisco, Srijan Sen, Ambuj Tewari

**Affiliations:** 1 Department of Statistics University of Michigan Ann Arbor, MI United States; 2 Department of Statistics and Data Science Carnegie Mellon University Pittsburgh, PA United States; 3 Eisenberg Family Depression Center University of Michigan Ann Arbor, MI United States; 4 Molecular and Behavioral Neuroscience Institute University of Michigan Ann Arbor, MI United States; 5 Department of Electrical Engineering and Computer Science University of Michigan Ann Arbor, MI United States

**Keywords:** privacy, data protection, machine learning, federated learning, neural networks, mobile health, mHealth, wearable electronic devices, differential privacy, privacy, learning, evidence, feasibility, applications, training, technology, mobile phone

## Abstract

**Background:**

Although evidence supporting the feasibility of large-scale mobile health (mHealth) systems continues to grow, privacy protection remains an important implementation challenge. The potential scale of publicly available mHealth applications and the sensitive nature of the data involved will inevitably attract unwanted attention from adversarial actors seeking to compromise user privacy. Although privacy-preserving technologies such as federated learning (FL) and differential privacy (DP) offer strong theoretical guarantees, it is not clear how such technologies actually perform under real-world conditions.

**Objective:**

Using data from the University of Michigan Intern Health Study (IHS), we assessed the privacy protection capabilities of FL and DP against the trade-offs in the associated model’s accuracy and training time. Using a simulated external attack on a target mHealth system, we aimed to measure the effectiveness of such an attack under various levels of privacy protection on the target system and measure the costs to the target system’s performance associated with the chosen levels of privacy protection.

**Methods:**

A neural network classifier that attempts to predict IHS participant daily mood ecological momentary assessment score from sensor data served as our target system. An external attacker attempted to identify participants whose average mood ecological momentary assessment score is lower than the global average. The attack followed techniques in the literature, given the relevant assumptions about the abilities of the attacker. For measuring attack effectiveness, we collected attack success metrics (area under the curve [AUC], positive predictive value, and sensitivity), and for measuring privacy costs, we calculated the target model training time and measured the model utility metrics. Both sets of metrics are reported under varying degrees of privacy protection on the target.

**Results:**

We found that FL alone does not provide adequate protection against the privacy attack proposed above, where the attacker’s AUC in determining which participants exhibit lower than average mood is over 0.90 in the worst-case scenario. However, under the highest level of DP tested in this study, the attacker’s AUC fell to approximately 0.59 with only a 10% point decrease in the target’s *R*^2^ and a 43% increase in model training time. Attack positive predictive value and sensitivity followed similar trends. Finally, we showed that participants in the IHS most likely to require strong privacy protection are also most at risk from this particular privacy attack and subsequently stand to benefit the most from these privacy-preserving technologies.

**Conclusions:**

Our results demonstrated both the necessity of proactive privacy protection research and the feasibility of the current FL and DP methods implemented in a real mHealth scenario. Our simulation methods characterized the privacy-utility trade-off in our mHealth setup using highly interpretable metrics, providing a framework for future research into privacy-preserving technologies in data-driven health and medical applications.

## Introduction

### Background

The rise of mobile health (mHealth) as an exciting and compelling health care paradigm has been unmistakable. As wearable devices continue to gain popularity and smartphone penetration continues to rise globally [1,2], opportunities for mHealth to make positive impacts on health care delivery and administration have proliferated. On the research side, the period between the years 2018 and 2020 has seen more mHealth-related publications than the previous years combined [3]. Meanwhile, the current global mHealth market is expected to experience annual growth of 11% for the better part of this decade [2].

However, widespread adoption of mHealth technologies may not occur until the challenge of user data privacy is overcome [4]. Recent polling has shown that the vast majority of Americans do not believe that the benefits of releasing personal data to private companies are worth the risks [5]. Research has also shown that privacy concerns directly affect the willingness of users to participate in mHealth systems, especially younger users [6] and users with diseases that carry social stigma [7]. Along with the increased frequency of cyberattacks on centralized data centers and IT infrastructure and the growing number of smart devices that use these facilities [8], the costs of insufficient security and privacy protection for future large-scale mHealth applications are abundantly clear.

The collection of privacy-enhancing tools spans the entirety of the data collection and use pipeline, including approaches such as data policies, encryption, access control, and secure multiparty computation [9]. In this study, we examined 2 popular algorithmic strategies (federated learning [FL] and differential privacy [DP]) and explored some of their shortcomings. Specifically, we demonstrated the trade-off between algorithmic utility and privacy protection when implementing these strategies in practical mHealth settings. We aimed to offer insights into the potential of these strategies to protect user data by constructing a simulated attack on a centralized server.

### FL Strategy

FL is a machine learning method used when data are distributed across independent devices (often referred to as clients). In the traditional centralized learning regime, a data collector interested in constructing a statistical model for answering queries about the data would first aggregate all the data onto a central server before performing model optimization on all data simultaneously. However, in the FL setting, all data remain with the individual client, and only the statistical model resides on the central server. Optimization of the FL model occurs in a distributed fashion, where copies of the model are sent to clients and model *updates* are aggregated from clients to the central server at each optimization step. Here, individual updates are calculated using only the data on the client device.

The exact implementation details of FL depend on the statistical model being constructed, but its main benefit over the centralized regime is that a security breach of the central server is far less serious. In the worst-case scenario, the attacker can only access historical communications between the central server and clients; none of which will contain any private user data. However, FL can reduce the utility of the central model [10] and slow optimization depending on the availability of individual clients. Nevertheless, initial research on FL in an mHealth context has shown that models trained in a federated manner have only minor performance costs compared with models trained centrally [11]. In this regard, FL would seem like a prime candidate for privacy protection in large-scale mHealth applications; however, we show that FL alone is not sufficient to defend against all privacy threats.

### DP Strategy

DP aims to overcome the problems associated with information leakage that occur whenever a query is conducted on a private data set. In short, if the answer to a query (eg, What is the average age of patients in XYZ hospital?) given a fixed data set is deterministic, a clever attacker could combine a series of queries with auxiliary information to infer private information about individual records in the data set. This includes whether a particular record is present in the data set (membership inference) or features associated with a particular record (data reconstruction).

DP protects against such attacks by adding a stochastic component to query outputs. For example, when the output is a continuous value, it is often accomplished by adding random noise to the answer. As there is no longer a deterministic mapping between data set properties and query outputs, the probability of an attacker successfully inferring private information is reduced. In conventional ε-δ DP, we say that a mechanism *M* that generates an output based on an input data set *D* is ε-δ differentially private if the following inequality holds:

*Pr[M (D_1) ∈ A] ≤ e^ε^ Pr[M (D_2) ∈ A] + δ* **(1)**

where *A* is any set of possible output values and *D_1_* and *D_2_* differ only in the presence or absence of a single record [12]. In other words, a differentially private construction provides strong bounds on the probability of a successful membership inference attack by enforcing that the model output is sufficiently close (defined by epsilon and delta) in distribution to what the model would have output if one user’s record was deleted from the data set.

As with FL, the exact implementation of DP depends on the nature of the queries made on a private data set. DP is commonly used to control information leakage that results from exposing the parameters of the models trained on private data. For example, Abadi et al [13] demonstrated how DP can be integrated into gradient descent algorithms for deep learning to achieve certain statistical guarantees of privacy. As with FL, the benefits of DP come at the cost of lower model utility, often embodied in lower model accuracy and longer optimization times.

### Previous Work

FL has widespread application in health care settings. Using electronic health records distributed across hospital systems, researchers have successfully developed federated models to predict heart-related hospitalizations [14], electrocardiogram classification [15], and clinical outcomes in patients with COVID-19 [16]. In addition to the results in a study by Liu et al [11], other studies have shown that FL can be applied to wearable sensor data in biomedical applications [17], a particular area of interest for mHealth research. Such studies generally find that the benefits of FL outweigh the costs.

Similarly, there have been many uses of DP in health care applications [18], including drug sensitivity prediction [19] and coronary heart disease diagnosis [20]. Other studies combined DP with FL to achieve enhanced privacy protection [21]. However, such studies have not demonstrated a universal rule for choosing the ε-DP parameter in any given application. Hsu et al [22] found that the optimal choices of ε in the existing literature span orders of magnitude depending on the context. Therefore, this study aimed to contribute to the DP literature by evaluating its efficacy in a novel health sensor data application.

Although quantifying model utility is straightforward in most cases, quantifying privacy protection is usually imprecise. The benefits of FL are clear at the conceptual level (eliminating risks associated with centralizing data in one location), but how this translates to a quantifiable increase in privacy protection is unclear. Although DP provides strong statistical guarantees tied to numeric parameters (such as in ε-δ DP), such guarantees only directly apply to a specific class of privacy attacks (membership inference attacks).

Attempts have been made to measure the effectiveness of FL and DP against other types of attacks, most notably property inference attacks that aim to uncover the private attributes of the training data. For instance, Naseri et al [23] constructed simulated privacy attacks against an image classifier trained on the Labeled Faces in the Wild data set. Even with the protection of FL and local DP, they found that these classifiers are still vulnerable to external property inference [23]. Melis et al [24] conducted similar experiments on both the Labeled Faces in the Wild data set and the Yelp reviews data set and arrived at a similar conclusion [24]. However, both papers limit the number of clients in their FL setup to no more than 30, which is far below the scale of mHealth applications intended for public use. Furthermore, it is unclear whether their findings for image and text classifiers extend to other domains and data types, such as time series sensor data collected from wearable health devices.

In this study, we measured the privacy-utility trade-off of FL and DP using wearable sensor data gathered from a large-scale clinical study of medical interns. We adopted an existing simulation-based methodology tailored to a realistic mHealth setup to understand how FL and DP might perform in this specific domain. To the best of our knowledge, this is the first study to (1) evaluate the effectiveness of FL and DP on a real-world health sensor data set, (2) use simulation-based methodology on time series sensor data at the scale of thousands of FL clients, and (3) characterize which FL clients are most at risk from these external attacks if privacy protection is insufficient.

## Methods

### Ethics Approval

The data used in this analysis come from the University of Michigan Intern Health Study, approved by the Institutional Review Boards of the University of Michigan Medical School (IRBMED), review number HUM00033029.

### Overview

We must construct models of a target system and an external attack to evaluate the strength of privacy protection mechanisms for guarding against actual privacy attacks. The *Target System* is a prediction model existing on a central server that implements FL and DP in an attempt to protect against privacy threats. The *External Attack* simulates an adversarial actor intent on uncovering private information about individuals in the *Target System*. Enacting the *External Attack* against the *Target System* allows us to assess the performance of the Target System in terms of model utility and privacy protection.

### Data: Intern Health Study

Data for this study were obtained from the 2017 to 2019 cohorts of the University of Michigan Intern Health Study (IHS) [25]. IHS aims to investigate the biological and genetic factors affecting the relationship between stress and depression. The study followed medical interns at several dozen facilities in the United States and China. In addition to providing demographic information at the beginning of the study, participants were asked to wear a Fitbit device during their internship and complete daily mood ecological momentary assessments (EMAs) through a mobile app.

The data originally covered 6660 registered participants with 1,241,629 daily sensor observations. After data cleaning (see Table 1 for details), our final data set contained 4274 participants and 596,585 daily sensor observations. Although the IHS data included information about participants’ medical internships (such as specialty), we excluded this information to better simulate an mHealth system that would be used with the general population. The pertinent demographic features of the data are given in Tables 1 and 2, whereas Table 3 shows the summary statistics for participant age, daily mood, and daily sensor data. Table 4 summarizes the degree of missingness for each sensor measurement for all the daily observations.

**Table 1:** Participants and sensor data by cohort year.

Cohort year	Participants (raw data; n=6660), n	Participants (clean data^a^; n=4274), n	Daily sensor observations (raw data; n=1,235,543^b^), n	Daily sensor observations (clean data; n=596,585), n
2017	2846	531	119,850	83,999
2018	2129	2098	576,535	277,754
2019	1685	1645	539,158	234,832

^a^Participants with no available sensor data and sensor observations not linked to the registered participants were removed. We also excluded 40 participants with invalid or missing age values, 2 participants with missing sex values, and 26 participants with missing ethnicity values. Sensor observations with no mood scores were excluded.

^b^This number is slightly less than the original data set size because of observations not linked to registered participants.

**Table 2:** Breakdown of participants by ethnicity and gender (clean data).

	Male (n=1896), n (%)	Female (n=2378), n (%)
Arab or Middle Eastern	40 (2.11)	37 (1.56)
Asian (eg, Indian or Chinese)	456 (24.05)	524 (22.04)
Black or African American	73 (3.85)	148 (6.22)
Latino or Hispanic	87 (4.59)	81 (3.41)
Multiracial	156 (8.23)	223 (9.38)
Native American	1 (0.05)	3 (0.13)
Other	10 (0.53)	8 (0.34)
Pacific Islander	1 (0.05)	0 (0)
White	1072 (56.54)	1354 (56.94)

**Table 3:** Descriptive statistics of daily sensor data and participant age (clean data).

	Values, mean (SD)	Values, median (range)
Participant age (years)	27.66 (2.62)	27 (17-47)
Mood ecological momentary assessment score	7.27 (1.63)	7 (1-10)
In-bed minutes	417.98 (137.59)	437 (4-1379)
Sleep minutes	372.11 (123.81)	389 (0-1228)
Active minutes	63.83 (92.6)	25 (0-1016)
Step count	8819 (4739)	8215 (1-70,138)
Resting heart rate (beats/min)	62.83 (7.63)	63 (39-100)

**Table 4:** Daily sensor data missingness (clean data).

	Days missing^a^, (n=596,585), n (%)	Days of data per participant, mean (SD)
In-bed minutes	259,633 (43.52)	78.84 (99.70)
Sleep minutes	259,633 (43.52)	78.84 (99.70)
Active minutes	351,181 (58.87)	57.42 (69.56)
Step count	186,189 (31.21)	96.02 (107.44)
Resting heart rate	323,722 (54.26)	63.84 (77.52)
Combined features^b^	428,692 (71.86)	39.28 (61.77)

^a^Missingness was measured relative to the baseline obtained after removing all days with missing mood scores and all participants with missing demographic features or no sensor data.

^b^A nonmissing day for this row is any day with all 5 sensor measurements recorded.

### Data Preprocessing

The most important step in preparing the IHS data for analysis was to deal with missing data. Although a typical medical internship lasted 12 months, the average participant in the study logged a bit more than 1 month (not necessarily continuous) of complete sensor data. Given our objective of building a real-world mHealth system, we could not exclude all observations with any missing features. Furthermore, exploratory tests demonstrated that neural network classifiers trained on only complete cases are not useful predictors of mood scores. Therefore, we imputed missing sensor features using 10 iterations of multivariate imputation by chained equations because of its flexibility and relatively low computational cost [26]. We acknowledge that true FL would require us to impute data locally at the participant level, but data availability considerations led us to adopt a centralized imputation approach.

Our feature set included the 9 features (cohort year, age, sex, ethnicity, and 5 sensor measurements) listed in the previous section, as well as 1-day and 2-day lags for each of the 5 sensor measurements and the mood score. Lags account for the possibility that certain sensor events (such as one night of poor sleep) may have a delayed impact on mood. In addition, we included 3 time-based features indicating the observation’s day of week, day of month, and day of year to account for unmeasured factors that may be correlated with time. Finally, we performed common preprocessing steps on our feature set, such as standardizing continuous features and splitting categorical features into component binary features.

### Target System Construction

In the Target System, the statistical models live on the central server and use data from individual IHS participant devices for training. We formulated both regression and classification tasks for the Target System and trained models for each using our sensor and demographic data. These 2 tasks provide robustness in assessing the effectiveness of our privacy protection measures. The regression task predicts mood scores on the 1- to 10-point scale, whereas the binary classification task predicts whether the user’s mood has improved from the previous day.

As we did not consider model interpretability in our analysis, we implemented neural networks for both tasks because of their flexibility and accuracy advantages over other machine learning methods. We also considered the fact that future mHealth systems open to the public are likely to collect data on a far larger scale than that obtained in the IHS. Although a full investigation of all possible model types for the Target System is outside the scope of this analysis, we presented comparisons with simpler linear methods (ordinary least squares for the regression task and logistic regression for the binary classification task) to justify our choice of neural networks. The details of the neural network implementation are provided in Multimedia Appendix 1. This paper reports the results from the regression task and leaves the results from the binary prediction task in Multimedia Appendix 2.

### External Attack: Underlying Assumptions

Several methods can be designed to compromise user privacy through the Target System. For example, attackers interested in whether a specific user or record appears in the Target System may mount a *membership inference attack*, whereas those interested in ascertaining certain statistical properties of the private training data (either globally or on a per-user basis) may mount a *property inference attack*. Other attackers may attempt *data reconstruction attacks*, which aim to reconstruct partial or complete records from the original training data [23]. Our attack model is derived from domain-specific considerations regarding the environment of mHealth applications, the privacy demands of system users, and the identities of possible attackers.

In mHealth systems containing a large portion of the general population, membership inference has limited value, and the reconstruction of a specific user’s data is quite difficult. Therefore, we assume that the attacker is interested in property inference on individual user data, particularly whether an IHS participant has an average daily mood EMA score higher than the global average daily mood EMA score (denoted hereafter by *mood status*). Such inferences are relatively easy to execute and could expose sensitive information about the participants; for instance, those with consistently low mood scores may be at a higher risk of depression or other mental disorders. Regardless of the attacker’s exact purposes, the mere possibility of a successful inference of mood status may significantly undermine public trust in the mHealth system.

The literature on privacy attacks provides 2 broad dimensions along which a privacy threat may be assessed based on the attacker’s resources. The first dimension is the attacker’s level of access to the Target System and any associated privacy protection systems. The literature often differentiates between *black box* model access, where the attacker is limited to viewing only the Target System’s model output for a given input, and *white box* access, where the attacker is able to view the model architecture and all related parameters, along with the details of any privacy protection mechanisms. The second dimension is the attacker’s ability to alter the model parameters for liking. Certain attacks can be implemented passively, meaning that the attacker can compromise user privacy by simply observing changes to the Target System’s statistical model that occur during training. Others require the attacker to actively influence the model parameters, usually by injecting customized training data into the system.

We assumed that the attacker has white box access to the central server of the Target System (including the statistical model and all communications with the server) but can only carry out privacy attacks passively. We also assumed that the attacker has no ability to access any live individual user devices in the Target System (any devices actively participating in model training). These assumptions match the *rogue employee* profile, an insider who is easily able to access confidential details about the Target System but would not be able to effect changes in model parameters without raising suspicion. This profile was selected to balance plausibility with preparation for a worst-case scenario. Although it is unlikely that any adversarial actor could influence the training process of the Target System, we believe that any serious actor would likely gain insider access.

Implicit in this assumption is the ineffectiveness of other commonly used privacy-preserving technologies outside FL and DP against this type of threat. We assumed that privacy policies and operational protocols would be of limited use against bad faith actors, whereas encryption and authentication technologies would not stop an attacker with insider access. This is not to say that such tools are not useful for ensuring user privacy and building trust. However, for the simplicity of this analysis, we assumed that such conventional methods are not effective and focus on the ability of FL and DP to stop such an attacker. We elaborate on the reasonableness of this assumption and implications for future research in the Discussion section.

### External Attack: Implementation

#### Overview

The implementation details of this attack follow those in the study by Melis et al [24]. The attack was conducted in the 3 stages described in the following sections. For this analysis, we assumed that the statistical model in the Target System is trained using FL and local DP, implying that individual user devices do not pool their data centrally and add noise to their gradient updates before sending them to the central server. Other than the gradient updates, all user data, including sensor measurements, demographic data, and mood EMA scores, remain strictly on individual user devices. Additional details can be found in the *Implementing FL and DP* section.

#### Stage 1: Accessing Target Model Parameters

**Figure 1:**
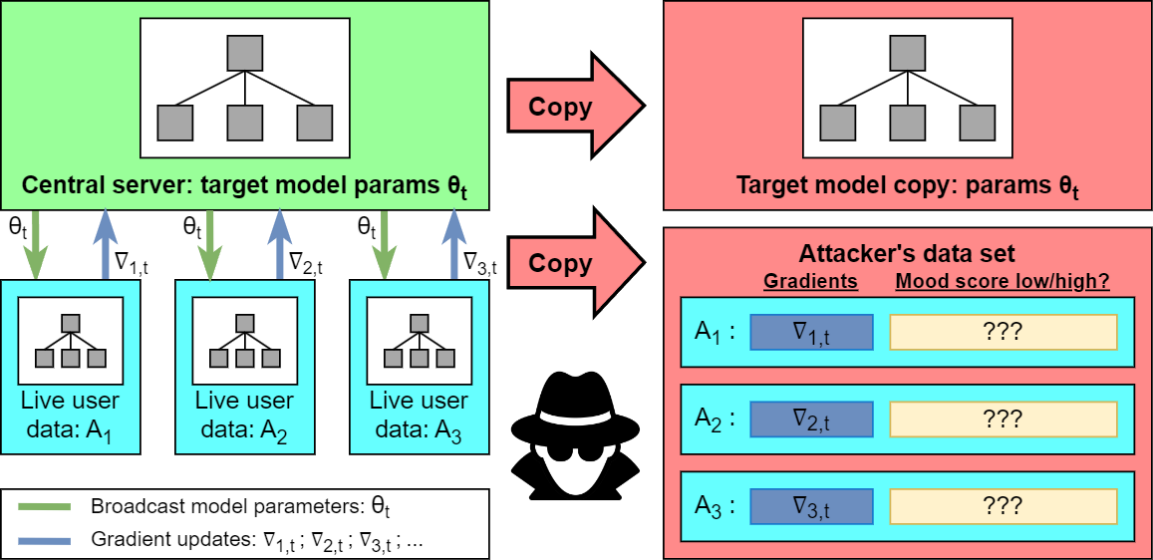
Attacker infiltration of central server.

Given that the attacker has full access to all information stored on the central server at training time, we assumed that they observe the Target System model parameters at time *t* (given by *θ_t_*) as well as the gradient updates (given by ∇_i,t_) with respect to *θ_t_* sent to the server from individual user devices *A_i_*. The relationship between these variables is shown on the left side of Figure 1.

The attacker first copies the entire Target Model (including parameters *θ_t_*) and the gradient updates ∇_i,t_. The attacker ultimately attempts to predict the mood status of each user *A_i_*. Note that under DP, the gradient updates ∇_i,t_ observed by the attacker would include any noise added to the true model gradient on the user device.

#### Stage 2: Constructing the Attacker’s Data Set

**Figure 2:**
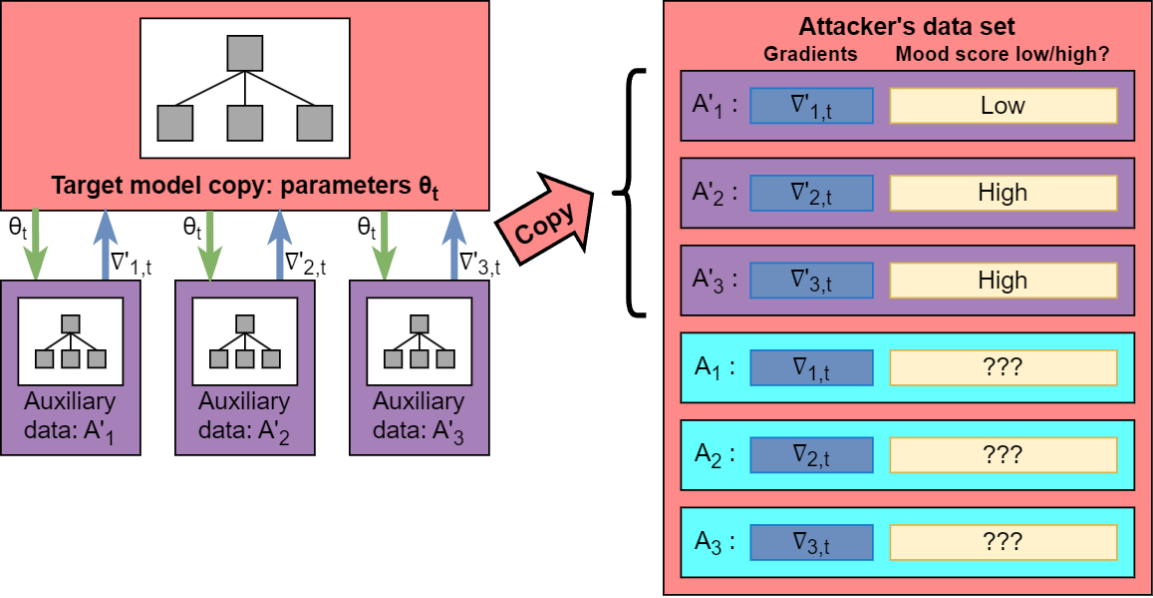
Constructing the attacker’s data set.

To predict the mood status of live users, the attacker requires an auxiliary collection of users for whom the mood status is known, and model gradients with respect to *θ_t_* can be calculated. We assumed that attackers could access such a data set because they already have access to the central server. These data could be sourced from central server databases containing raw data for pilot users, a small number of previously compromised user devices, an externally published data set, or even users working in collusion with the attacker. The process of using these data to construct the attacker’s data set is shown in Figure 2. In particular, each user in the auxiliary data has a known mood status and generates a gradient ∇’_i,t_ with respect to the same Target Model parameter *θ_t_*. This gradient contains the same amount of noise as the observed gradient updates from live users because we assumed that the parameters used for privacy protection in the Target System are stored on the central server. We reasoned this assumption is feasible for systems implementing FL and DP because they cannot broadcast the relevant parameters to all users while hiding them from adversaries with access to the central server. This increases the likelihood of a successful inference relative to if the noise parameter is unknown.

#### Stage 3: Mood Status Prediction

**Figure 3:**
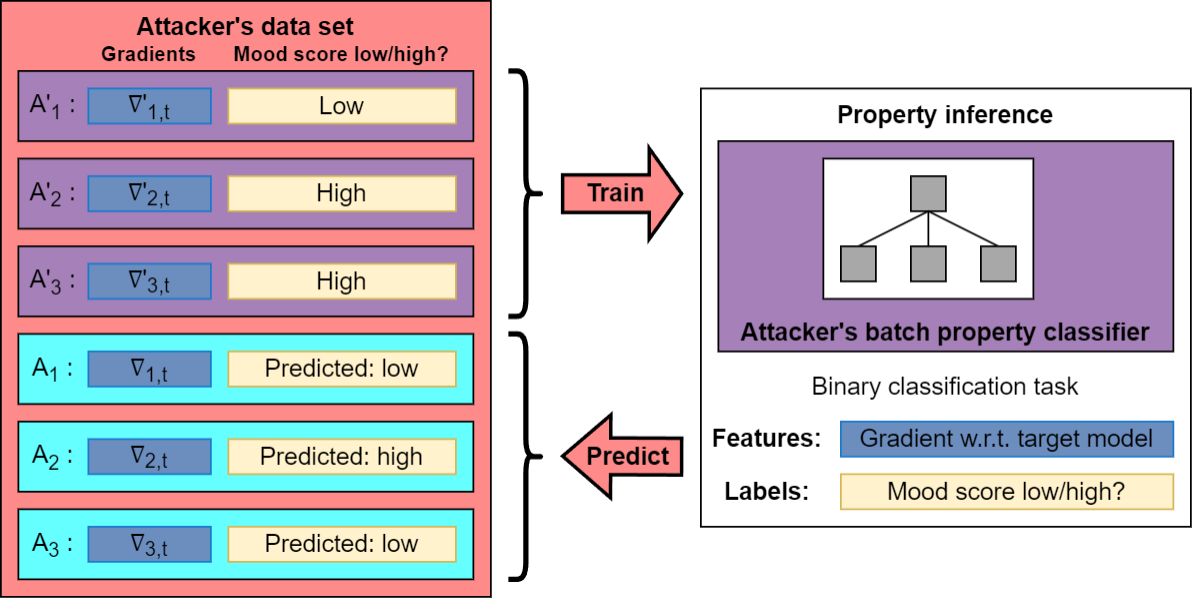
External attack training and final inference.

Finally, the records in the attacker’s data set for which mood status is observed can be used to train a binary batch property classifier, which predicts mood status (labels) from the provided gradients (features). This process is illustrated in Figure 3. Any machine learning model can be used in this step, but we adopted a neural network approach following the example in a study by Melis et al [24]. The details of the implementation are provided in Multimedia Appendix 1.

### Implementing FL and DP

**Figure 4:**
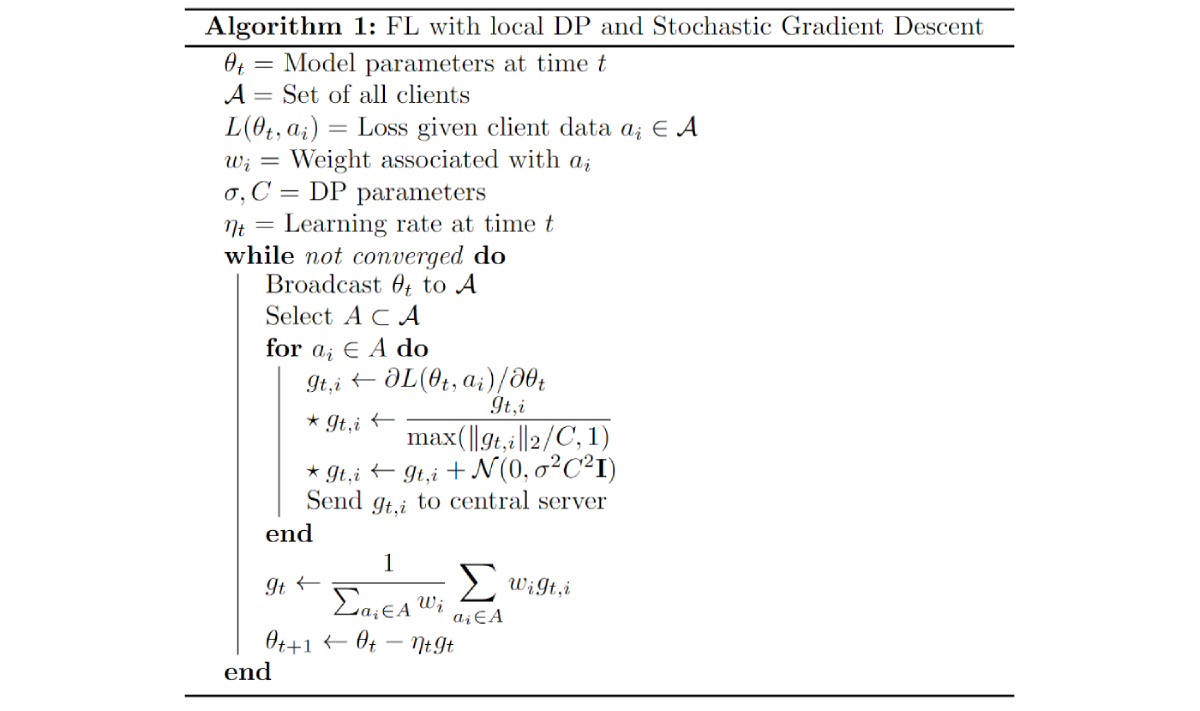
Pseudocode for privacy protection implementation. DP: differential privacy; FL: federated learning.

Our proposed defense against external attacks uses local DP techniques in conjunction with FL to mask the information present in gradient updates communicated to the central server. The specific implementation follows the procedure given in Naseri et al [23]. The general algorithm is illustrated in Figure 4.

Our implementation of FL randomly sampled 30% of the study participants in each iteration to participate in the corresponding model update (set *A*). Our procedure is very similar to that of vanilla FL and differs only in the second and third steps (indicated with a star) of the inner loop, which are described as follows:

1. Clip each computed gradient to have *L_2_* norm of at most *C*.
2. Add gaussian noise with mean 0 and variance *σ^2^C^2^* to each gradient component independently.

The first step ensures that the signals within the gradient updates do not overpower the noise, whereas the second step provides the stochastic component that addresses information leakage resulting from deterministic gradient calculations. For this analysis, we set *C=1* for all cases and varied the *σ* parameter to achieve different privacy protection levels.

Much of the existing literature on DP reported explicit ε and *δ* values based on the training process used for the Target System (including any noise added) as well as the properties of the training data. Although it is certainly possible to report our level of privacy protection in terms of ε (after fixing a value for *δ*), we chose to report *σ* (the noise scale) directly for a variety of reasons. The ε-*δ* guarantees of DP did not directly apply to property inference and hence our simulation-based approach to measuring the true level of privacy protection. The other main roles of ε and *δ* (communicating the degree of perturbation applied to the training process) could also be served by reporting the more interpretable noise scale parameter *σ*. We varied only the *σ* parameter in the course of the analysis, creating an effective one-to-one correspondence between ε and *σ* values. Given that the main stakeholders for large-scale mHealth systems were health care practitioners and the public, we believed that the interpretability of our analysis should take precedence over theoretical rigor.

Other parameters remain identical to those outlined in Multimedia Appendix 1 for the Target System.

### Performance Versus Privacy Protection: A Simulation Approach

#### Overview

**Figure 5:**
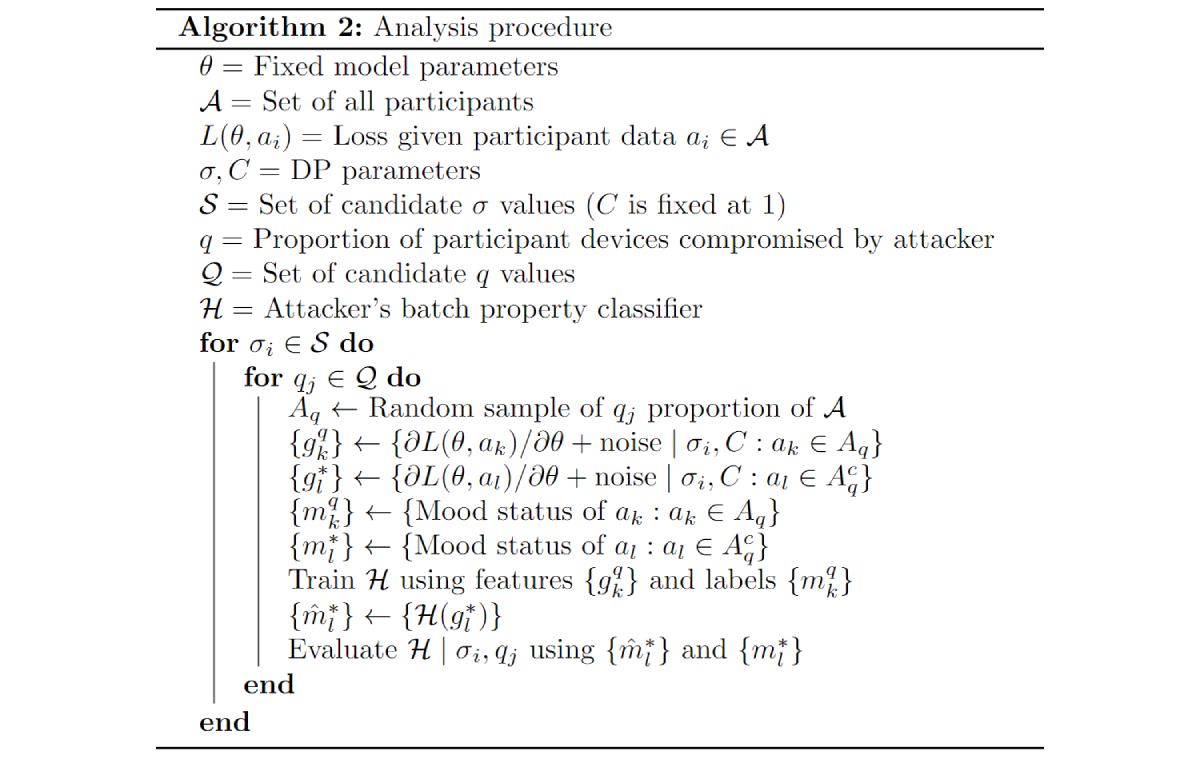
Pseudocode for our assessment of external attack effectiveness. DP: differential privacy.

In general, it is difficult to quantify the effectiveness of an *External Attack* against the *Target System*; therefore, we used a simulation-based approach to report results for several different attacker settings. We varied σ, the noise parameter in constructing the stochastic gradients, to test different levels of privacy protection. We also modeled differences in the attacker’s access to auxiliary user data by changing *q*, the proportion of IHS study participants with compromised devices whose data are available to the attacker. Figure 5 shows the basic simulation setup used in the analyses.

#### Measuring Costs of Privacy Protection

Higher levels of privacy protection generally hinder the ability of the Target System to train the model effectively (because the gradients have more added noise). We measured this cost in terms of the training time and final model utility for different values of σ. To facilitate comparability, we initialized the Target Model identically for each case and seeded all stochastic components in the training process (selection of clients and network dropout) identically.

#### Measuring Effectiveness of Privacy Protection

We measured the effectiveness of our privacy protection procedure by the *External Attack*’s ability to infer the mood status of noncompromised users. Designating a fixed subset of such users as a test set, we recorded the maximum test area under the curve achieved by the attacker’s classifier *at any point during its training process (up to 600 epochs)*. Although the attacker theoretically does not have access to the testing labels, this procedure provides an experimental upper bound on their performance. We also calculated the model sensitivity and positive predictive value (PPV) as more easily interpretable metrics. All metrics are calculated for each choice of σ and *q*. To facilitate comparability, all evaluations were performed with the attacker using the same Target Model parameters produced after 20 training epochs to generate their gradient data set. All the other stochastic components were seeded identically for each evaluation.

## Results

### Target Model Training Metrics

**Figure 6:**
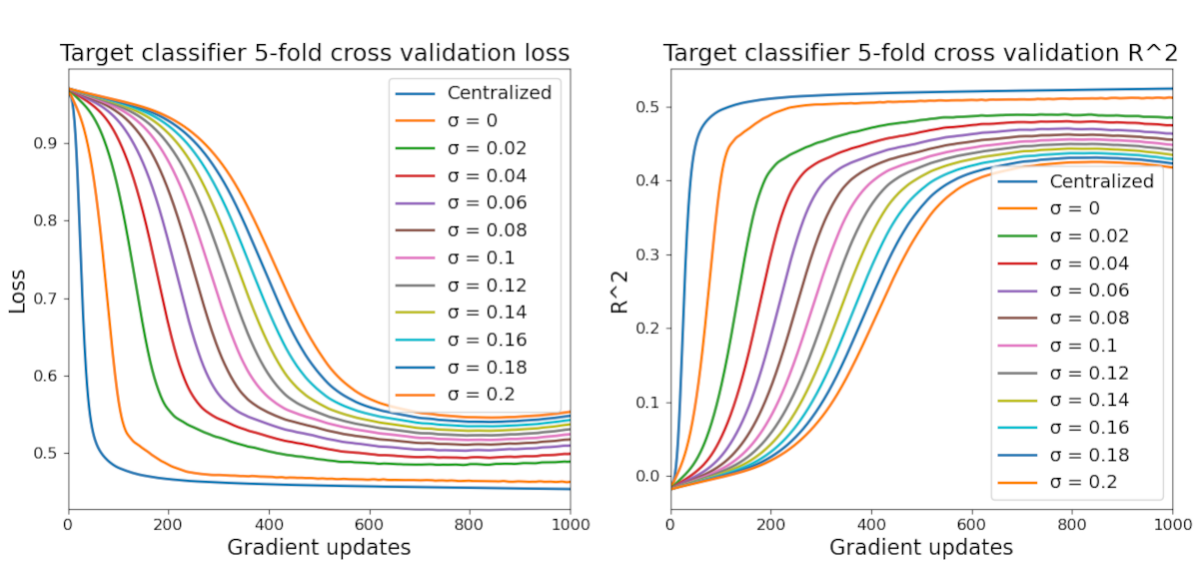
Target system model performance. In all simulations, training runs for at most 1000 epochs.

**Figure 7:**
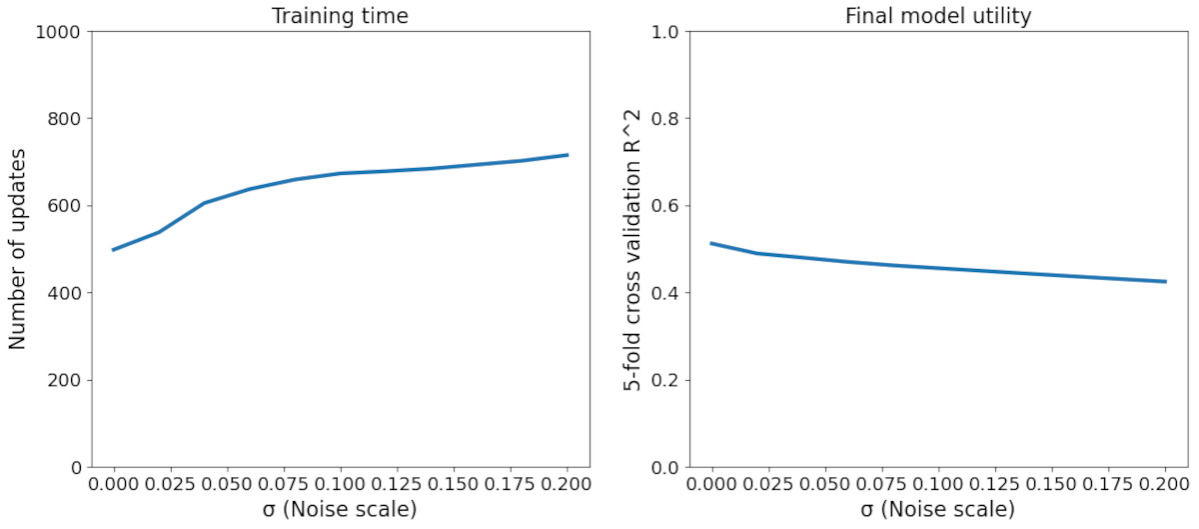
Training time or final model utility versus noise scale.

Figure 6 plots the Target System’s model loss and accuracy metrics over each gradient update. Training progress under the conventional centralized training protocol is also plotted for reference. A σ value of 0 denotes FL with no DP implementation. We saw a clear effect of both FL and DP on the training process for the Target System’s statistical model. Relative to the centralized protocol, FL leads to a noticeable increase in training time, but the final model accuracy seems to be very similar (at least within the given training window). The addition of noise via DP seems to substantially affect both training time and final model accuracy. Interestingly, training loss seems to begin increasing at some point in the training process when additional noise is added, potentially indicating shortcomings in conventional optimization techniques when using both FL and DP. Figure 7 shows the relationship between training time, final model utility, and noise scale, where training time is defined as the number of gradient updates needed for model loss to fall within 1% of the minimum loss over 1000 updates.

### Privacy Protection Metrics

**Figure 8:**
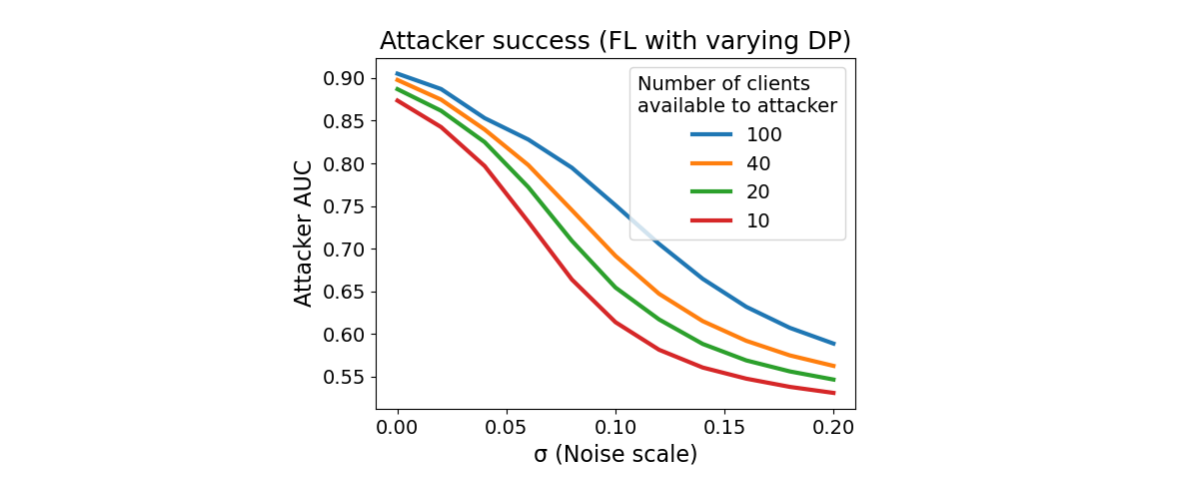
Attacker success versus noise scale. AUC: area under the curve; DP: differential privacy; FL: federated learning.

**Figure 9:**
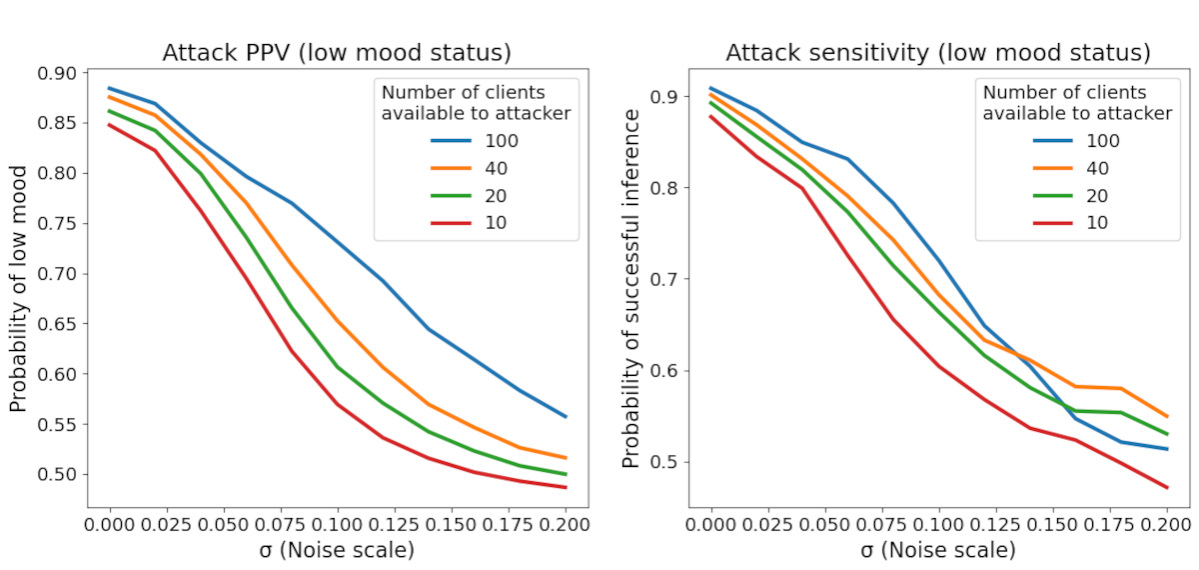
Attack PPV or sensitivity versus noise scale. PPV: positive predictive value.

Figure 8 shows the success of the *External Attack* in inferring a participant’s mood status based on the participant’s observed gradient to the fixed *Target System* model parameters. Immediately, we noted that FL alone (0 noise) is insufficient to protect against this type of attack. Even if the attacker has access to labeled data for only 10 participants (of 4274 total participants), they can successfully infer the mood status of a large majority of all other participants. However, adding noise to the gradient updates significantly decreases the attacker’s performance even when they have access to a large amount of labeled training data. Figure 9 shows similar results for the attack’s PPV and sensitivity, given that a low mood status is viewed as a positive test result.

Combined with the training metrics from the previous section, we can begin to assign concrete trade-offs between model utility and privacy protection in the context of this particular Target Model. For example, if the attacker has access to 100 participants’ labeled training data, setting the noise scale to 0.1 increases the training time by 26.2%, decreases the final model *R*^2^ by 11.5%, and decreases the privacy attack’s PPV by 17% relative to FL with no additional noise. Increasing the noise scale to 0.2 increases training time by 40.7%, decreases final model *R*^2^ by 17.3%, and decreases the PPV of privacy attack by 36.4% compared with FL with no noise.

### Vulnerability Metrics

This section analyzes the benefits of our privacy protection procedure for various subgroups within our data. Although we should strive to maximize privacy protection within reason for all members of the population, it is instructive to examine who is most at risk from this privacy threat and hence who benefits the most from these privacy protection mechanisms.

**Figure 10:**
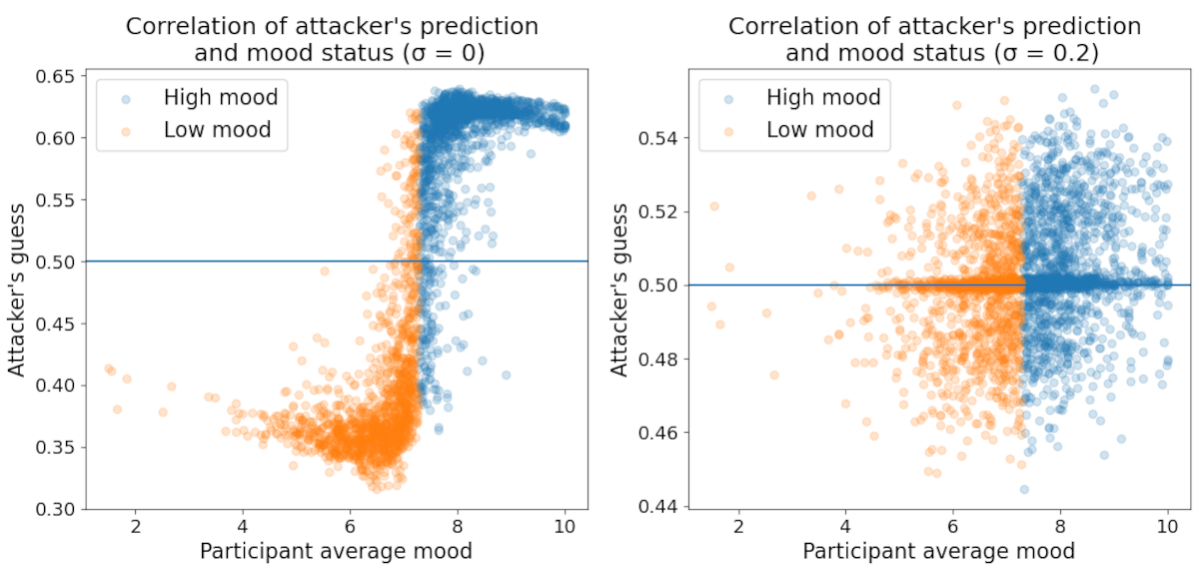
Attacker’s predicted mood status versus participant average mood for different differential privacy (DP) noise parameters.

Figure 10 shows the correlation between the attacker’s prediction of a particular participant’s mood status versus the participant’s actual mood status. The y-axis shows the attacker’s predicted probability of each participant having a high mood status, where values above 0.5 indicate participants ultimately classified as having a high mood status. As expected, there was a strong positive association between the attacker’s prediction and the participant’s actual average mood when no additional noise was added to the FL. This indicates that participants with average mood scores that were much lower than the global average were simultaneously at a much higher risk of a successful inference attack. As privacy concerns are usually greatest for those with sensitive health conditions, this further underscores the insufficiency of FL alone. Fortunately, the addition of sufficient noise can eliminate this correlation, neutralizing privacy risks regardless of the participant’s mood tendencies. Similar analyses of privacy risk across gender, ethnicity, and age groups did not yield noteworthy findings. The associated figures can be found in Multimedia Appendix 1.

## Discussion

### Principal Findings

Our results showed that FL combined with DP substantially reduced the attacker’s ability to infer the participant mood status in our simulation. The 3 attacker success metrics we report (area under the curve, PPV, and sensitivity) all approach 0.5 with increased noise, meaning that the attacker only performs slightly better than a random guess in these situations. In addition, the attacker does not perform better when classifying those with extreme mood scores, protecting those who may be more vulnerable to an attack. This protection results in a decrease in the utility of the target model, but even in the worst-case scenario, *R*^2^ decreases by only 17.3% compared with FL without noise.

In contrast to the studies by Naseri et al [23] and Melis et al [24], both of which found that FL and DP are insufficient to protect against this type of attack in other domains [23,24], we found that FL and DP could potentially provide a reasonable trade-off when used in a large-scale mHealth system based on health sensor data. A natural next step is to understand the reasons for this difference on a theoretical basis and explore how FL and DP fare in domains outside of those mentioned in this analysis. Nevertheless, these results underscored our belief that context is crucial in privacy research; there is no universal approach that will work in every setting.

Our analysis addresses the challenge of quantifying and communicating the level of protection offered by privacy-preserving methods. We narrowed the scope of the *External Attack* and adopted a simulation-based approach to produce metrics that are both meaningful and easy to interpret. Our results underscore two important principles for future work on privacy protection: (1) FL, despite its simplicity and broad acceptance, is insufficient to protect against advanced privacy attacks on its own and (2) mHealth users whose health indicators deviate strongly from those of the general population (ie, those who most require privacy protection) are at a higher risk under our threat model. We must evaluate and improve existing tools for privacy protection and ensure that new approaches consider the individual needs and vulnerabilities of system users.

### Limitations of the Analysis

It is possible that our implementation of the property inference attack may not exploit sufficient weaknesses in our Target System setup or our implementation of FL with local DP may not be perfectly optimized for our data set. These issues would result in model metrics that overstate the efficacy of our privacy protection measures or the cost of privacy protection. Future research could establish more credibility for implementing FL with DP by verifying our results using modified target systems and attacks. Experiments with variations of the FL and DP algorithms could also provide more favorable trade-offs against some or all external attacks.

Our methods are also predicated on specific assumptions about the capabilities and resources of the attacker. We assumed the failure of more conventional privacy-preserving technologies to stop attackers. Although this may not be true in all real-world scenarios, the number of high-profile cybersecurity breaches in recent years justifies preparing for the worst-case scenario. Given sufficient incentive, it is likely that bad actors would attempt to procure the access and resources required to bypass conventional privacy measures and carry out our proposed attack. Future research should examine how FL and DP interact with other conventional privacy measures, especially if other technologies can compensate for the weaknesses of FL and DP.

Missing data imputation is another area for future research on FL and DP implementation. Our imputation procedure is one of the main threats to the validity of our results, and it seems likely that future mHealth studies will suffer from similar problems. Although our analysis does not devote attention to a rigorous solution to this problem, we acknowledged that imputation methods deserve the same scrutiny for privacy risks as the other tools used for mHealth research.

It is important to note that our simulation-based approach to measuring privacy protection sacrifices some statistical rigor in favor of interpretability. Unlike many DP implementations, we cannot guarantee the maximum bounds on the attacker’s probability of successful inference. It is not entirely clear how the mechanics of this attack change based on the Target System’s model architecture (neural network or otherwise), Target System’s classification task, attacker’s resources, attacker’s intended inference task, or properties of the underlying population. We hope that this work provides the foundation for the future development of numerical methods to approximate protection levels over a broader range of models and populations or even theoretical bounds on the likelihood of successful property inference in mHealth systems.

### Conclusions

It is our sincere hope that mHealth research will continue to generate robust tools for privacy protection along with novel statistical methodologies and technical improvements. The weaknesses of FL clearly demonstrated the dangers of complacency: threats will continue to evolve and our privacy protection technologies cannot fall behind. As mHealth applications continue to scale, safeguarding public trust must remain a top priority for researchers and practitioners alike.

## Appendix

Multimedia Appendix 1 Additional details and results for the regression task.

Multimedia Appendix 2 Results from the binary classification task.

## Abbreviations

AUC: area under the curve
DP: differential privacy
EMA: ecological momentary assessment
FL: federated learning
IHS: Intern Health Study
mHealth: mobile health
PPV: positive predictive value

## Data Availability

The data sets generated and analyzed during this study are not publicly available because of considerations regarding participant privacy but are available from the corresponding author on reasonable request.

The computer code that supports the findings of this study is available on reasonable request from the corresponding author, AS. The code is not publicly available because of considerations of participant privacy. However, a version of the code used in this study was developed for use with the publicly available Wearable Stress and Affect Detection (WESAD) data set [27]. The code and instructions for downloading the WESAD data and running the experiments are available on the web [28].
