# Supplementary material for: Exploring the Relationship Between Privacy and Utility in Mobile Health: Algorithm Development and Validation via Simulations of Federated Learning, Differential Privacy, and External Attacks
Source: J Med Internet Res. 2023 Apr 20;25:e43664. doi: 10.2196/43664 (PMC10160928; doi:10.2196/43664)
Supplement: Multimedia Appendix 1 [file jmir_v25i1e43664_app1.docx]

# Appendix I: Additional Details/Results for the Regression Task

For the regression task in the Target System, we use a multilayer perceptron architecture for our neural networks with 256 nodes on the first hidden layer, 128 nodes on the second hidden layer, and 64 nodes on the third hidden layer. Leaky ReLU activation with slope 0.01 and dropout of 0.2 is applied between layers, except before the output layer. The networks are trained using the Adam optimizer with learning rate 0.0001, batch size 256, and Mean Squared Error (MSE) loss. Hyperparameters are tuned using 5-fold cross validation.

Holding training/validation splits and features constant, we find that this neural network architecture slightly outperforms Ordinary Linear Regression (OLS) in terms of 5-fold CV R^2^. OLS achieves an R^2^ of approximately 0.51, while the neural network is able to achieve approximately 0.56 for the same metric.

For the mood status prediction in the External Attack, we use a neural network classifier with 24 nodes in the first hidden layer, 20 nodes in the second hidden layer, and 16 nodes in the last hidden layer. Batchnorm, Leaky ReLU activation with slope 0.01, and dropout of 0.2 is implemented between layers, except before the output layer. The model is optimized using the Adam optimizer with learning rate 0.001 and weight decay 0.15 (since there are a large number of components in gradient updates to the Target Model). Hyperparameters are optimized using 5-fold cross validation.

Figure 11 shows the External Attack’s sensitivity when the attacker has access to data for 100 IHS participants, split by mood status and age. There seem to be differences across age, although overall trends are hard to identify.


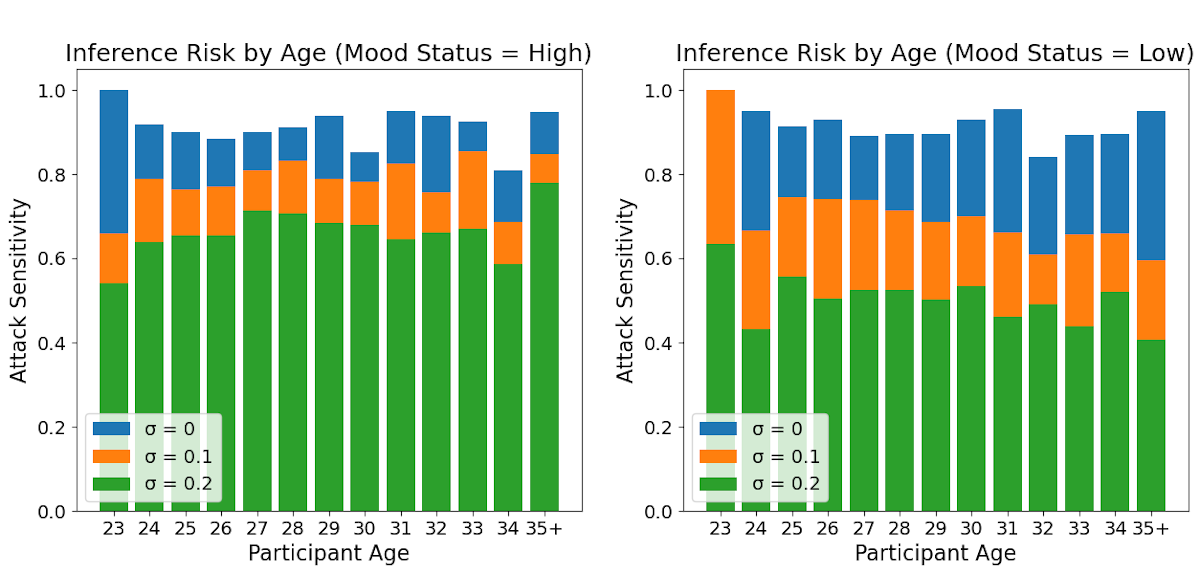


Figure 11: Attack Sensitivity by Age and Mood Status

Figure 12 shows the same data split by participant sex. There seem to be no significant differences between male and female participants.


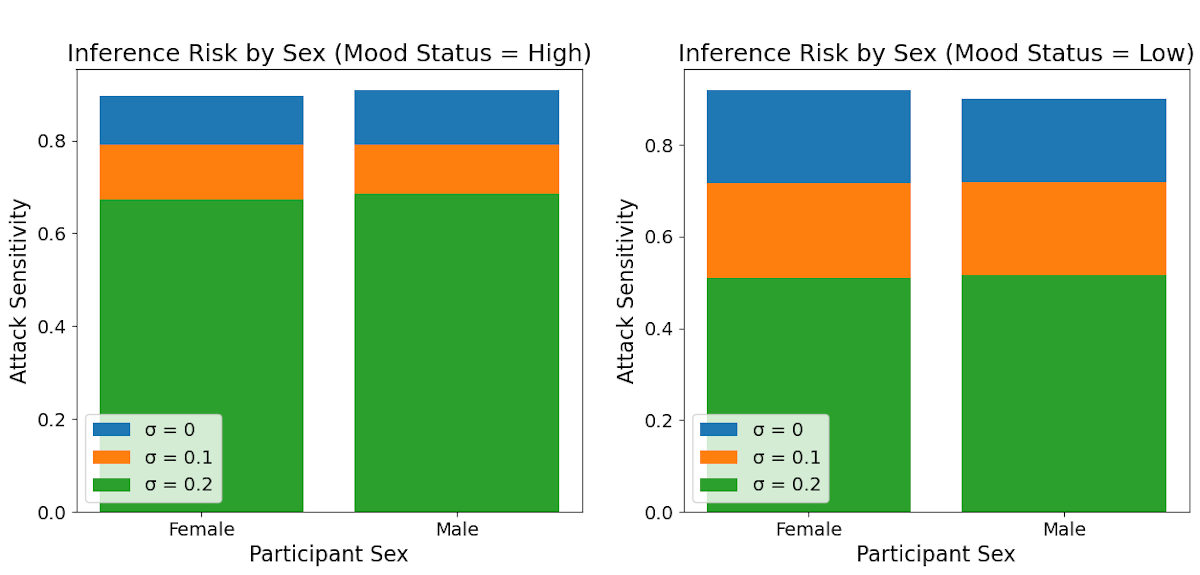


Figure 12: Attack Sensitivity by Sex and Mood Status

Figure 13 shows the same data split by participant ethnicity. For those with low mood status, adding additional noise to the Target System gradient updates seems to have heterogeneous effects on attack sensitivity, although small participant counts for some ethnic groups complicate drawing conclusions from these results.


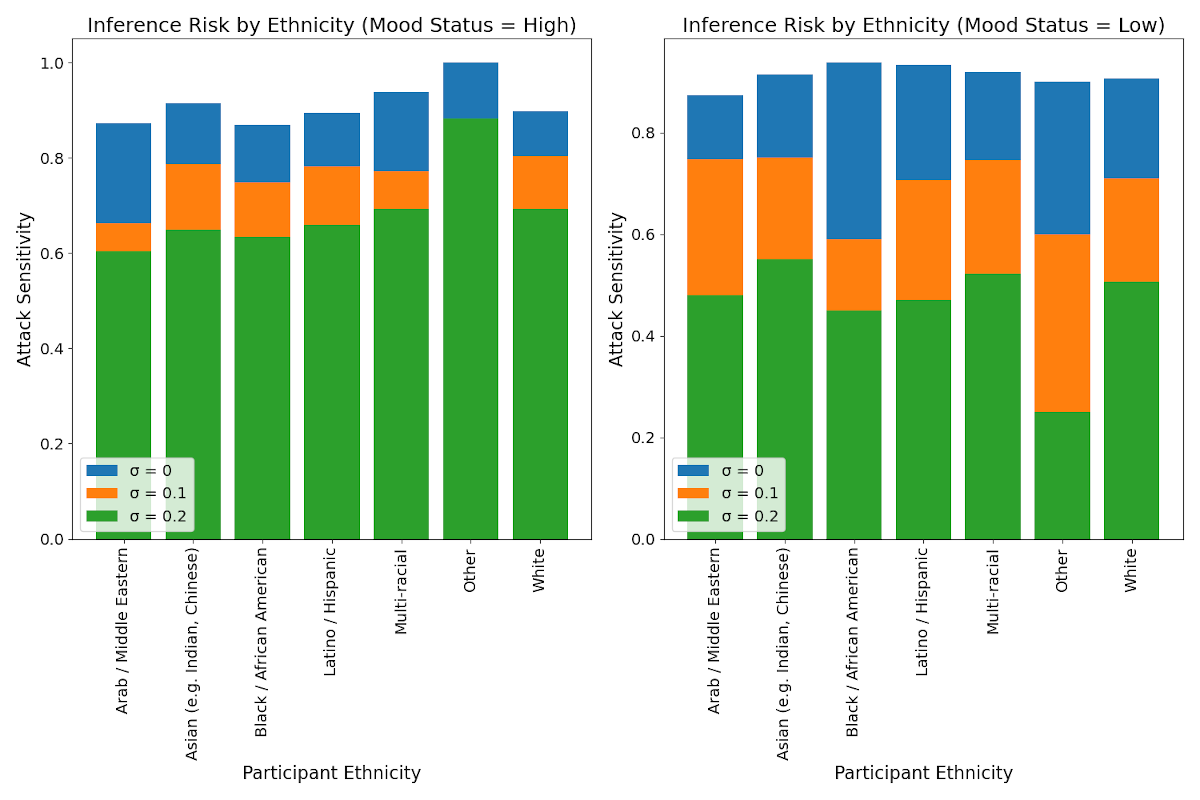


Figure 13: Attack Sensitivity by Ethnicity and Mood Status
