# Supplementary material for: Exploring the Relationship Between Privacy and Utility in Mobile Health: Algorithm Development and Validation via Simulations of Federated Learning, Differential Privacy, and External Attacks
Source: J Med Internet Res. 2023 Apr 20;25:e43664. doi: 10.2196/43664 (PMC10160928; doi:10.2196/43664)
Supplement: Multimedia Appendix 2 [file jmir_v25i1e43664_app2.docx]

# Appendix II: Results from the Binary Classification Task

For the classification task in the Target System, we use the same structure and training procedure for our neural networks as the one outlined in **Appendix I** for the regression task, except we replace MSE loss with Binary Cross Entropy (BCE) loss. The construction of the External Attack does not change from the details given in **Appendix I**.

Holding training/validation splits and features constant, we find that this neural network architecture slightly underperforms Logistic Regression in terms of 5-fold CV AUC. Logistic Regression achieves an AUC of approximately 0.78, while the neural network is able to achieve approximately 0.74 for the same metric. This difference could be partially attributed to the difference in complexity between the regression task and the binary classification task. In order to facilitate comparisons between tasks, we reuse the same neural network architecture between tasks, but it may not be optimal for the binary classification task. However, this difference in performance is not significant enough to detract from the main findings of the analysis, especially since the scale of data present in future mHealth systems for public use is likely to be much greater than that present in this analysis. Such a scale would warrant the use of more complex machine learning models like neural networks over simpler linear methods.

Under the binary classification task for the Target System, the attacker’s success rate in identifying private data attributes is over 80% in the worst case. However, under the highest level of DP tested in this paper, the attacker’s success rate falls to around 54.6% with only a 3 percentage point decrease in model accuracy and an apparent 92% increase in model training time.

Figure 14 plots the Target System model loss and accuracy metrics over each gradient update. Training progress under the conventional centralized training protocol is also plotted for reference. A *σ* value of zero denotes FL without any additional DP. We see very similar trends to those in the regression task, although we note that the model did not seem to converge within 1,000 epochs of training for the centralized regime and most values of the noise scale. This may affect our measurement of training times according to the stated definitions of convergence in the main body of the paper.


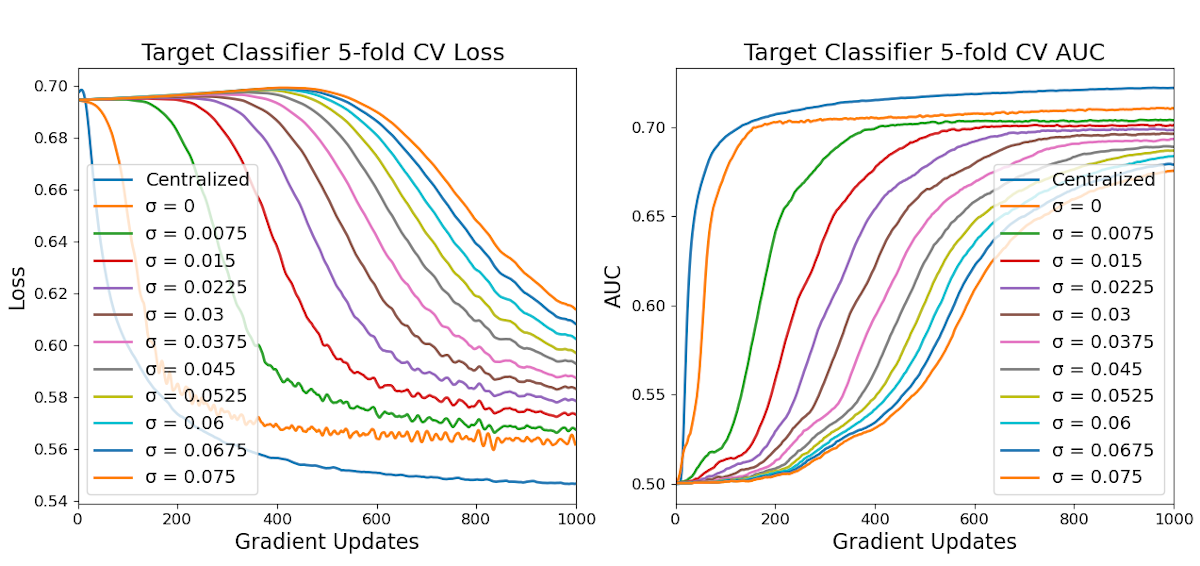


Figure 14: Target System Model Performance (Binary Classification Task)

Figure 15 shows the relationship between training time/final model utility and noise scale. We observe similar results as those generated in the regression task.


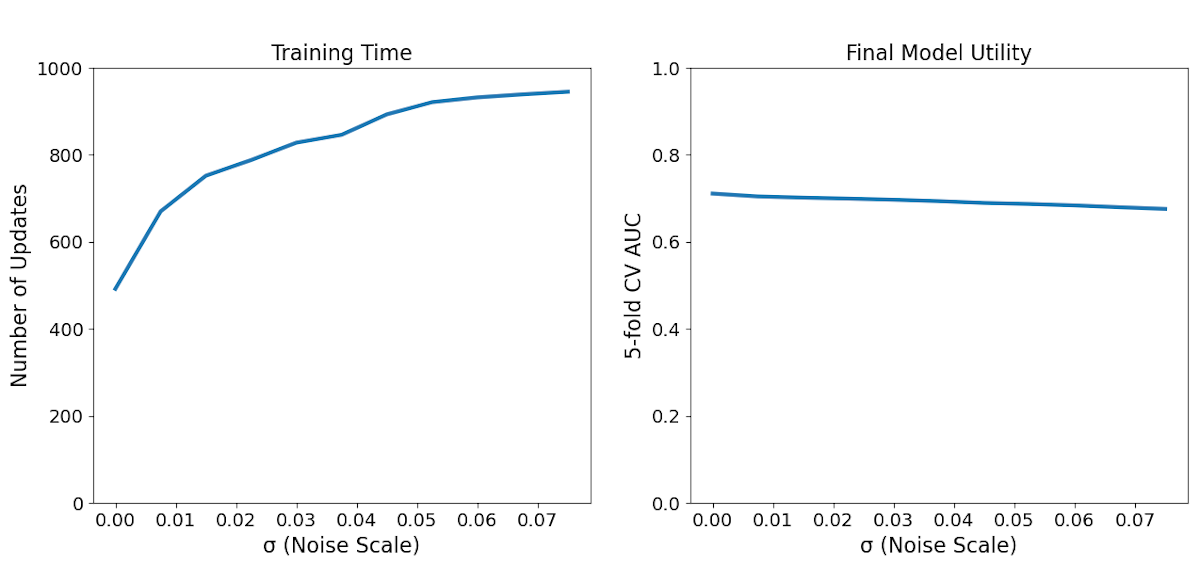


Figure 15: Training Time/Final Model Utility vs. Noise Scale (Binary Classification Task)

Figure 16 shows the External Attack’s success in inferring a particular participant’s mood status based on their observed gradient to the fixed Target System model parameters. We observe similar trends as those found in the regression task.


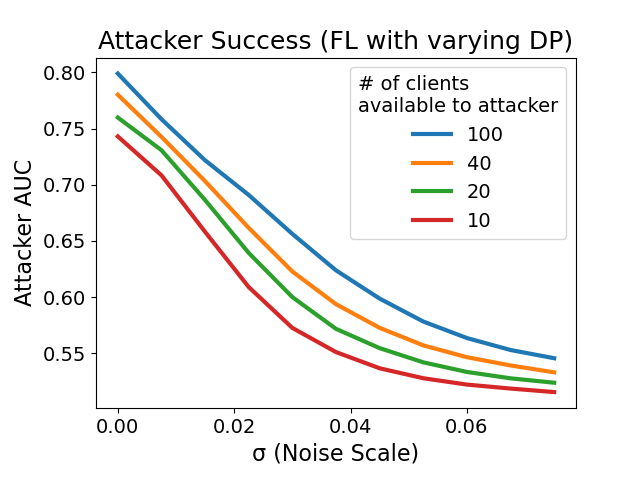


Figure 16: Attacker Success vs. Noise Scale (Binary Classification Task)

Figure 17 shows the property inference attack’s positive predictive value and sensitivity, given that a low mood status is viewed as a “positive test result”. We find similar results to the regression task for PPV, but we observe that trends for sensitivity across the noise scale and number of participants available to the attacker are less stable than those present in the results for the regression task. Specifically, we observe that sensitivity seems to increase at higher noise scales, although the results from attack AUC and PPV seem to suggest that the attack model may be attempting to compensate for higher noise by simply classifying more participants as “low” mood status overall.


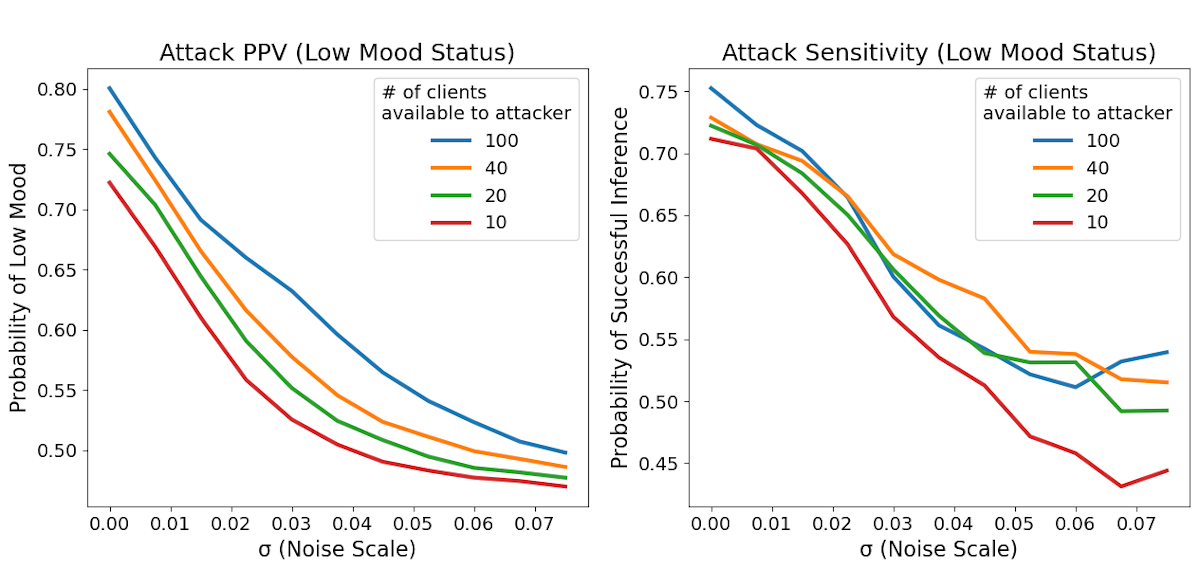


Figure 17: Attack PPV/Sensitivity vs. Noise Scale (Binary Classification Task)

Figure 18 shows the correlation between the attacker’s prediction of a particular participant’s mood status versus the participant’s actual mood status. We observe similar trends as those found in the results for the regression task.


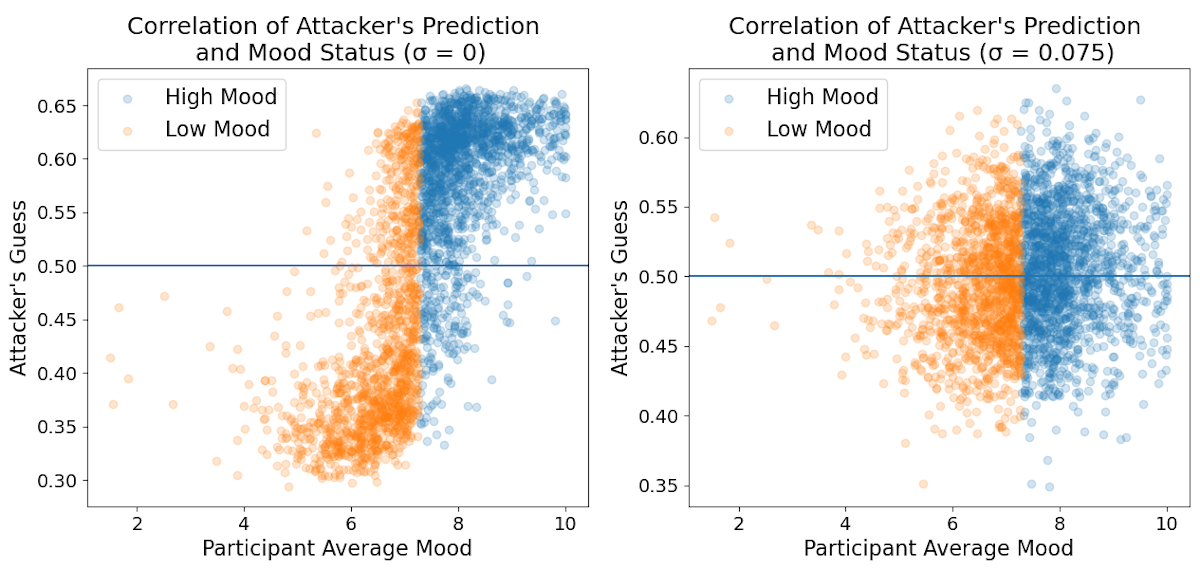


Figure 18: Attacker’s Predicted Mood Status vs. Participant Average Mood for Different Noise Levels (Binary Classification Task)

Figure 19 shows the External Attack’s sensitivity when the attacker has access to data for 100 IHS participants, split by mood status and age. There seem to be differences across age, although overall trends are hard to identify. We note that for some age groups in the low mood status participant block, adding noise actually slightly increases the attack sensitivity. This may be a result of the External Attack’s batch property classifier overfitting to its training data for certain demographic groups.


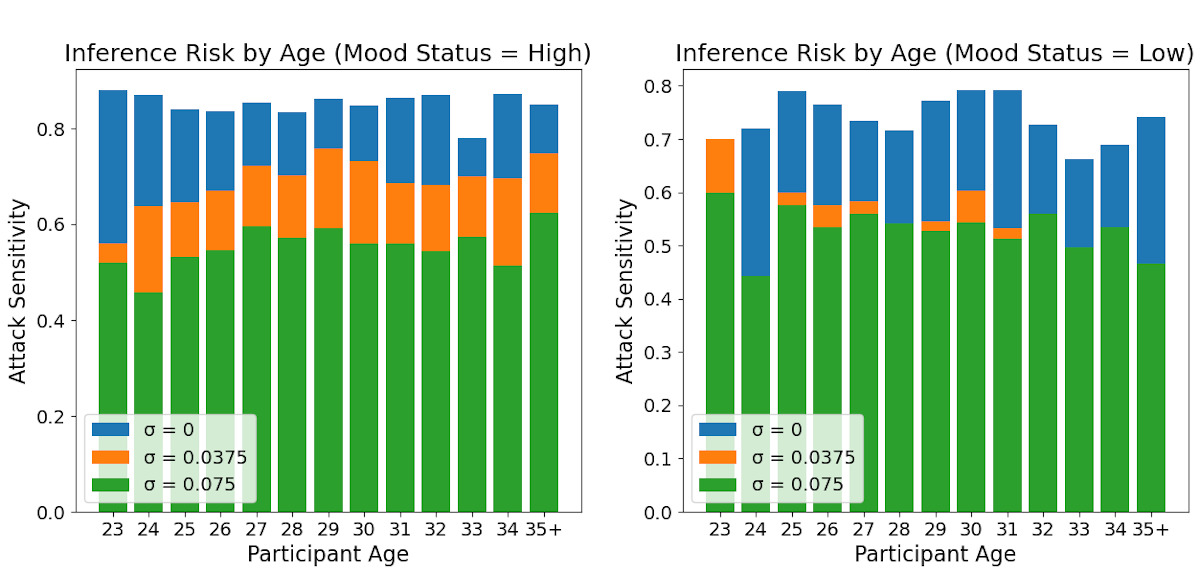


Figure 19: Attack Sensitivity by Age and Mood Status (Binary Classification Task)

Figure 20 shows the same data split by participant sex. There seem to be no significant differences between male and female participants.


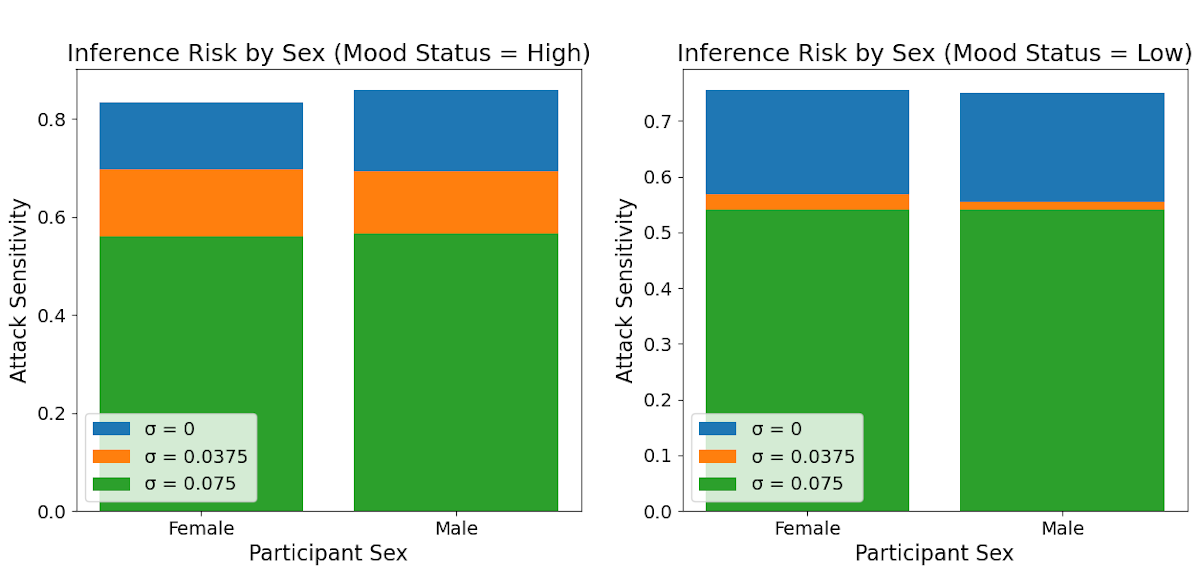


Figure 20: Attack Sensitivity by Sex and Mood Status (Binary Classification Task)

Figure 21 shows the same data split by participant ethnicity. For those with low mood status, adding additional noise to the Target System gradient updates seems to have heterogeneous effects on attack sensitivity, although small participant counts for some ethnic groups complicate drawing conclusions from these results.


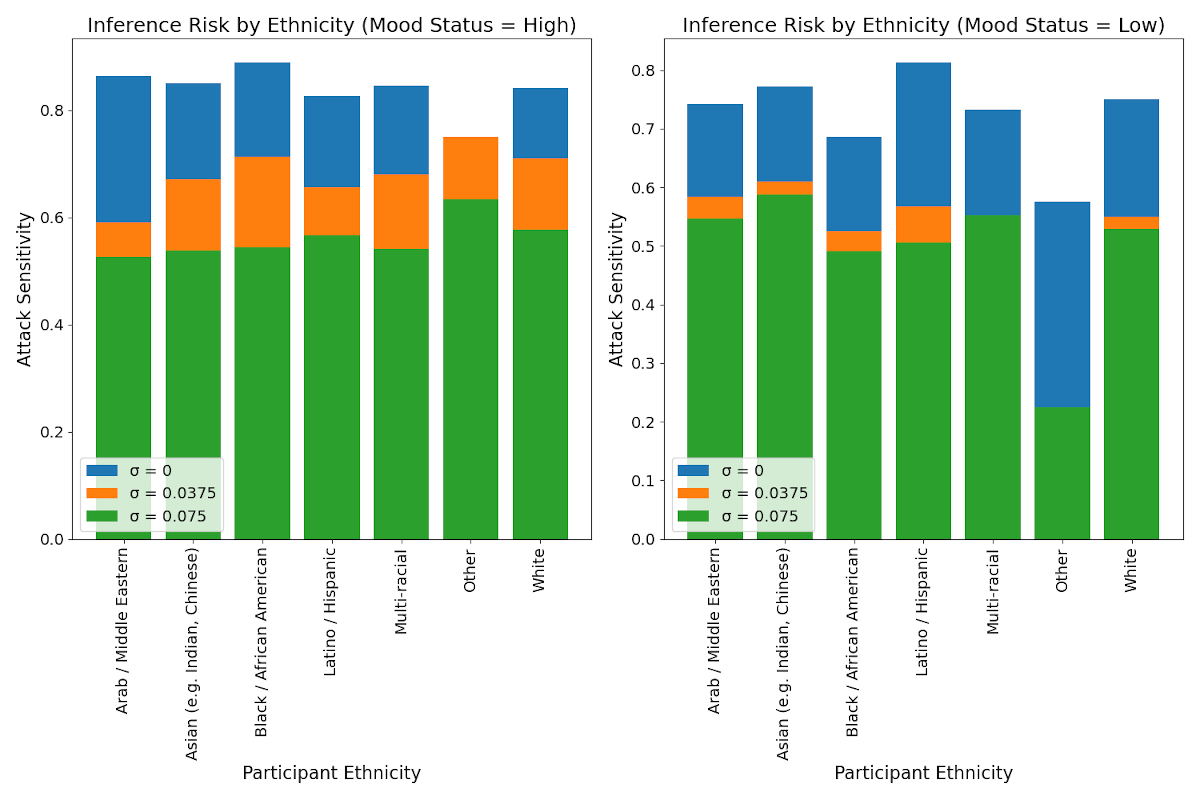


Figure 21: Attack Sensitivity by Ethnicity and Mood Status (Binary Classification Task)
